# Scientific Advances in Diabetes: The Impact of the Innovative Medicines Initiative

**DOI:** 10.3389/fmed.2021.688438

**Published:** 2021-07-06

**Authors:** Maria de Fátima Brito, Carla Torre, Beatriz Silva-Lima

**Affiliations:** ^1^Faculty of Pharmacy, University of Lisbon, Lisbon, Portugal; ^2^Laboratory of Systems Integration Pharmacology, Clinical & Regulatory Science–Research Institute for Medicines (iMED.ULisboa), Lisbon, Portugal

**Keywords:** innovative medicines initiative, diabetes, complications of diabetes, personalized medicine, type 2 diabetes, type 1 diabetes

## Abstract

Diabetes Mellitus is one of the World Health Organization's priority diseases under research by the first and second programmes of Innovative Medicines Initiative, with the acronyms IMI1 and IMI2, respectively. Up to October of 2019, 13 projects were funded by IMI for Diabetes & Metabolic disorders, namely SUMMIT, IMIDIA, DIRECT, StemBANCC, EMIF, EBiSC, INNODIA, RHAPSODY, BEAT-DKD, LITMUS, Hypo-RESOLVE, IM2PACT, and CARDIATEAM. In general, a total of €447 249 438 was spent by IMI in the area of Diabetes. In order to prompt a better integration of achievements between the different projects, we perform a literature review and used three data sources, namely the official project's websites, the contact with the project's coordinators and co-coordinator, and the CORDIS database. From the 662 citations identified, 185 were included. The data collected were integrated into the objectives proposed for the four IMI2 program research axes: (1) target and biomarker identification, (2) innovative clinical trials paradigms, (3) innovative medicines, and (4) patient-tailored adherence programmes. The IMI funded projects identified new biomarkers, medical and research tools, determinants of inter-individual variability, relevant pathways, clinical trial designs, clinical endpoints, therapeutic targets and concepts, pharmacologic agents, large-scale production strategies, and patient-centered predictive models for diabetes and its complications. Taking into account the scientific data produced, we provided a joint vision with strategies for integrating personalized medicine into healthcare practice. The major limitations of this article were the large gap of data in the libraries on the official project websites and even the Cordis database was not complete and up to date.

## Introduction

Innovative Medicines Initiative (IMI) is a unique pan-European public and private partnership that pioneered large-scale open collaborations between large pharmaceutical companies, small and medium-sized enterprises, public authorities (including regulators), organizations of patients, academia, and clinical centers to throw bottlenecks in research and development (R&D) of new effective and safer medicines (1).

To implement the Innovative Medicines Initiative, the European Commission and the European Federation of Pharmaceutical Industries and Associations (EFPIA) hold joint responsibility for creating and operating a new non-profit international organization (1).

IMI aims to accelerate the discovery and development of more effective vaccines, medicines, and treatments with fewer side-effects, especially in areas where there is an unmet medical or social need. IMI intends to implement patient centered projects, prompting the patient access to innovative pharmaceutical options (1, 2). This initiative provide socio-economic benefits and contribute to the health of European citizens, minimize duplication of work at different organizations, increase competitiveness, and help to establish Europe as the most attractive and competitive site for innovation (1, 2).

The first programme of IMI (IMI1) was created by Council Regulation (EC) n.° 73/2008, of 20th December 2007. The overall aim was to support pre-competitive pharmaceutical research and development, through the funding of innovative patient-centered projects for the research of European health priorities defined by the World Health Organization (WHO) (3). IMI1 programme was based on four strategic interdependent areas (Four-pillars) namely Safety, Efficacy, Knowledge Management, and Education and Training (1). The vision of this programme consisted on the creation of new scientific knowledge and capabilities/techniques to support the ability to identify a lack of efficacy or safety quickly in all stages of the medicine development process, even when a potential medicine has promising pre-clinical data (1). In addition, IMI1 programme intended to support the benefit-risk assessment conducted by the regulatory authorities (1, 2).

For this initiative, the budget committed was €2 billion (2, 4). During the execution period of IMI1 programme (2008 to 2013), eleven calls for proposals were released, which resulted in 59 funded-projects (4, 5).

The success of the IMI1 programme prompted the European Commission and the European Federation of Pharmaceutical Industries and Associations to take the commencing of initiating a second IMI programme (IMI2) under the Horizon 2020 vision of “improve the health and well-being of populations, reduce health inequalities, and ensure sustainable people-centered health systems” (5). Innovative Medicines Initiative 2 Joint Undertaking was established by Regulation (EU) n.° 557/2014, 6th of May (6). The major research axes recognized for IMI2 were: target & biomarker identification, innovative clinical trial paradigms, innovative medicines, and patient tailored adherence programmes (5). This programme ran from 2014 to 2020 and the budget committed was up to €3.276 billion, half funded by the European Comission and the other part from EFPIA.

Diabetes mellitus (DM) is one of the eleven priority diseases addressed by IMI1 and IMI2 programmes in the Strategic Reseach Agenda. This is a chronic metabolic disorder characterized by a defined phenotype (hyperglycemia accompanied by greater or lesser impairment in the metabolism of carbohydrates, lipids, and proteins), triggered by either lack of insulin secretion or decreased sensitivity of the tissues to insulin (7–9). Worldwide, a majority of diabetic patients (80–90%) have type 2 diabetes (T2D) and 5–10% type 1 diabetes (T1D) (8).

In 2014, WHO estimated that the prevalence of diabetes could reach more than 20% of the world's population within the next 20 years (8, 10, 11). Besides, the diabetes-associated mortality rate has been increasing, being the seventh leading cause of death in 2016 (12), and the disease and its acute and chronic complications represent a major economic burden on the global healthcare system and the wider global economy (5). For all the factors, previously presented, WHO considered this disease as the pandemic of the 21st century (8).

With the purpose of slowing the increasing prevalence, decreasing the mortality rate and diminishing the economic burden of diabetes and its related complications, IMI focused on projects aimed at understanding T1D and T2D, developing new precision medicines, identifying better patient-focused outcome measures for diagnosis, treatment selection and prognosis of T1D, T2D, and complications of diabetes, as well as promoting better lifestyle management and adherence to prescribed medicines (1, 5).

IMI1 programme funded six projects in the Diabetes & Metabolic disorders field, namely: Surrogate markers for micro- and macro-vascular hard endpoints for innovative diabetes tools (SUMMIT), Improving beta-cell function and identification of diagnostic biomarkers for treatment monitoring in diabetes (IMIDIA), Diabetes research on patient stratification (DIRECT), Stem cells for biological assays of novel medicines and predictive toxicology (StemBANCC), European Medical Information Framework (EMIF), and European Bank for induced pluripotent Stem Cells (EBiSC).

In IMI2 programme, until October of 2019, seven projects were supported in the area of Diabetes & metabolic disorders (13). These projects were: Translational approaches to disease modifying therapy of type 1 diabetes: an innovative approach toward understanding and arresting type 1 diabetes (INNODIA), Assessing risk and progression of prediabetes and type 2 diabetes to enable disease modification (RHAPSODY), Biomarker enterprise to attack DKD (BEAT-DKD), Liver Investigation: Testing Marker Utility in Steatohepatitis (LITMUS), Hypoglycaemia–redefining solutions for better lives (Hypo-RESOLVE), Investigating mechanisms and models predictive of accessibility of therapeutics into the brain (IM2PACT), and Cardiomyopathy in type 2 diabetes mellitus (CARDIATEAM).

Of the 13 projects, one was targeted to type 1 diabetes—INNODIA, three to type 2 diabetes—DIRECT, EMIF, and RHAPSODY, four to complications of diabetes—SUMMIT, BEAT-DKD, LITMUS, Hypo-RESOLVE, and CARDIATEAM, and the remaining four were scientific-oriented—StemBANCC, EBiSC, IMIDIA, and IMI2PACT. A more detailed description of the projects and its objectives is available in Supplementary Material section.

A total of €447.249.438 was mobilized for Diabetes (€253.865.866 from IMI1 and €193.383.572 from IMI2), however there has not been a systematization of scientific production by the IMI-funded projects.

The purpose of this literature review was to summarize the project results of IMI1 and IMI2 programmes into the major research axes of IMI2 programme and propose a joint vision model including the data collected into two inter-dependent paths, one scientific-oriented and the other medical-oriented.

## Materials and Methods

The data sources used in this review were the IMI website, the official project websites, contact with the project coordinators and co-coordinators, and the CORDIS (The Community Research and Development Information Service) database. From IMI website it was collected the project's start and end date, the grant agreement number, the contributions, and the coordinators and co-coordinators' e-mail addresses. The aim of each project was retrieved from its official website. The sources of the publications were the project's official website, CORDIS database, and the contact via e-mail with the coordinators and co-coordinators (Table 1).

**Table 1:** Summary of sources and publications included in this study.

**Projects**	**Articles obtained by contact via e-mail**	**Publications retrieved from the project's website**	**Articles collected on CORDIS database**
SUMMIT	–	52	42
IMIDIA	–	13	16
DIRECT	25	NA	NA
StemBANCC	–	91	31
EMIF	–	47	0
EBiSC	–	6	9
INNODIA	–	47	32
RHAPSODY	–	20	19
BEAT-DKD	–	52	38
LITMUS	–	0	0
Hypo- RESOLVE	0	0	0
IMI2PACT	–	0	0
CARDIATEAM	–	0	0

*NA, Not applicable*.

The contacts with the coordinators and co-coordinators were conducted in January of 2019 and for non-respondents, a recall in February of 2019. This step was performed for all projects.

The literature research on the project's websites and the CORDIS databases was conducted from February 2019 to October 2019. In October 2019, a new consultation was conducted on the IMI website, and the new funded projects (IMI2PACT and CARDIATEAM) were included.

For SUMMIT's project, a total of 98 citations were screened, 52 from the SUMMIT's website and 46 from the CORDIS database. A total of 67 references were excluded: (i) duplicates−29, (ii) book chapters−2, (iii) not access to the full text−7, (iv) the publication's objective was not related to diabetes mellitus or its complications−9, and (v) the publication's achievements did not allow to induce a scientific advance in Diabetes field (e.g., state of the art, outdated information, the article's data don't address an objective of the IMI2 programme)−20. For this project, a total of 31 articles were included.

For IMIDIA's project, a total of 29 citations were screened, 13 from the IMIDIA's website and 12 from CORDIS database. A total of 11 references were excluded: (i) duplicates−5, (ii) book chapters−1, (iii) the publication's objective was not related with diabetes mellitus or its complications−3, and (iv) the publication's achievements did not allow to induce a scientific advance in Diabetes field (e.g., state of the art, outdated information, the article's data don't address an objective of the IMI2 programme)−2. For this project, a total of 18 articles were included.

For DIRECT's project, a total of 25 citations were screened on the list of publications sent by the project coordinator. Since this list is not available online (either on the project's website or on CORDIS database), we present it in the Supplementary Material section. A total of nine references were excluded: (i) duplicates−1, (ii) not access to the full text−2, and (iii) the publication's achievements did not allow to induce a scientific advance in Diabetes field (e.g., state of the art, outdated information, the article's data don't address an objective of the IMI2 programme)−6. For this project, a total of 16 articles were included.

For StemBANCC's project, a total of 122 citations were screened, 91 from the StemBANCC's website and 31 from CORDIS database. A total of 103 references were excluded: (i) duplicates−30, (ii) book chapters−1, (iii) the publication's objective was not related to diabetes mellitus or its complications−71, and (iv) the publication's achievements did not allow to induce a scientific advance in Diabetes field (e.g., state of the art, outdated information, the article's data don't address an objective of the IMI2 programme)−1. For this project, a total of 19 articles were included.

For EMIF's project, a total of 165 citations were screened, all from the EMIF's website. A total of 136 references were excluded: (i) duplicates−1, (ii) article's exclusion criterion was the presence of diabetes−1, (iii) the publication's objective was not related with diabetes mellitus or its complications−120, and (iv) the publication's achievements did not allow to induce a scientific advance in Diabetes field (e.g., state of the art, outdated information, the article's data don't address an objective of the IMI2 programme)−14. For this project, a total of 29 articles were included.

For EBiSC's project, a total of 15 citations were screened, six from the EBiSC's website and 9 from CORDIS database. A total of 14 references were excluded: (i) the publication's objective was not related to diabetes mellitus or its complications (e.g., state of the art, outdated information, the article's data don't address an objective of the IMI2 programme)−14. For this project, a total of one article was included.

For INNODIA's project, a total of 79 citations were screened, 47 from the INNODIA's website and 32 from CORDIS database. A total of 34 references were excluded: (i) duplicates−32, (ii) not access to the full text−1, and (iii) the publication's achievements did not allow to induce a scientific advance in Diabetes field (e.g., state of the art, outdated information, the article's data don't address an objective of the IMI2 programme)−11. For this project, a total of 35 articles were included.

For RHAPSODY's project, a total of 39 citations were screened, 20 from the RHAPSODY's website and 19 from CORDIS database. A total of 28 references were excluded: (i) duplicates−21, (ii) book chapters−1, and (iii) the publication's achievements did not allow to induce a scientific advance in Diabetes field (e.g., state of the art, outdated information, the article's data don't address an objective of the IMI2 programme)−6. For this project, a total of 11 articles were included.

For BEAT-DKD's project, a total of 90 citations were screened, 52 from the BEAT-DKD's website and 38 from CORDIS database. A total of 65 references were excluded: (i) duplicates−40, (ii) the publication's objective was not related to diabetes mellitus or its complications−10, and (iii) the publication's achievements did not allow to induce a scientific advance in Diabetes field (e.g., state of the art, outdated information, the article's data don't address an objective of the IMI2 programme)−15.

No results were identified in LITMUS, Hypo-RESOLVE, IMI2PACT, and CARDIATEAM projects.

**Figure 1:**
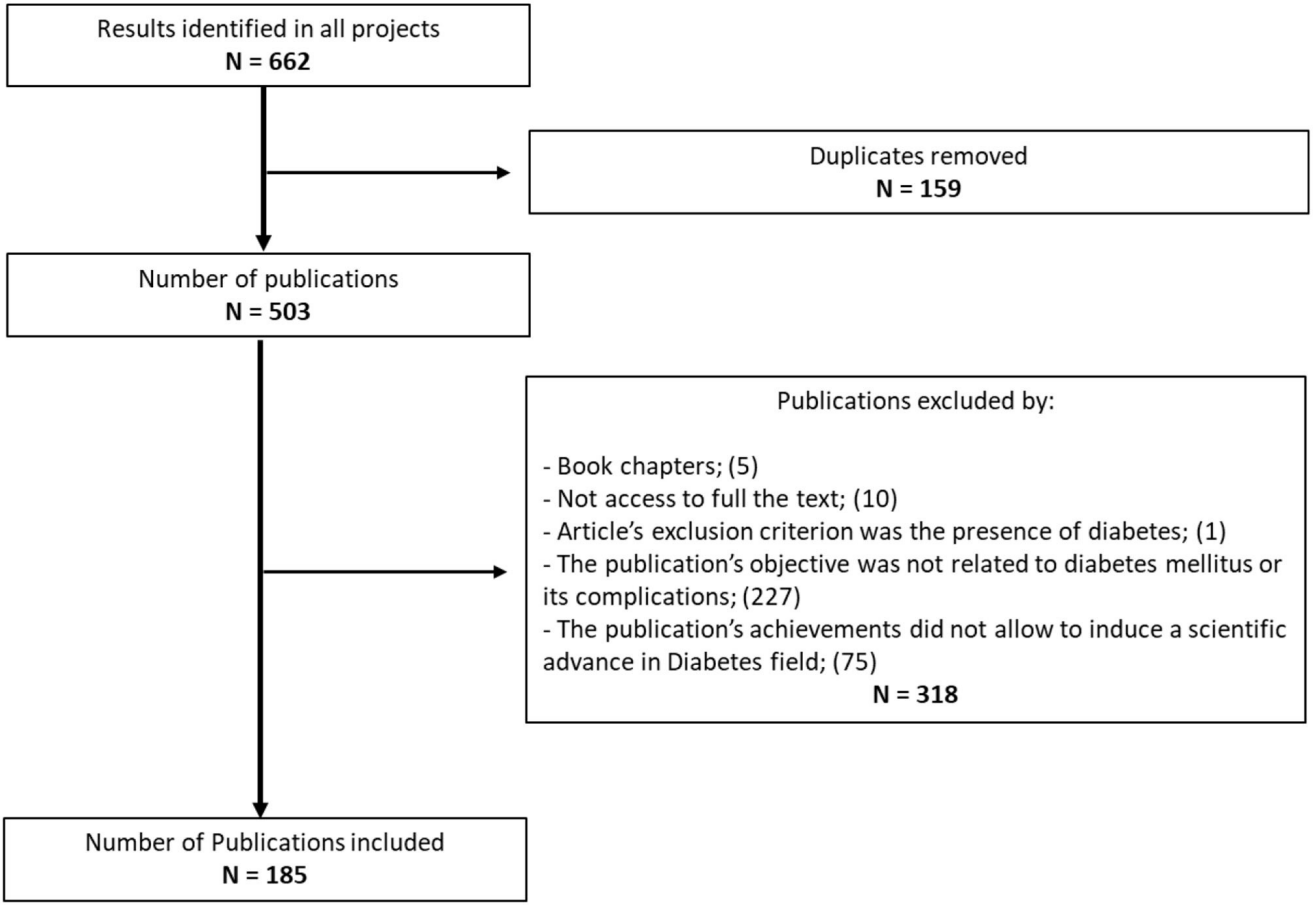
Flowchart of literature search.

The search and screening processes are summarized in Figure 1.

The results gathered in the literature review were integrated into the axes presented by the IMI2 programme, namely target & biomarker identification, innovative clinical trials paradigms, innovative medicines, and patient-tailored adherence programmes.

The data collected was organized according to the objectives established for each axis by the Strategic Research Agenda (SRA).

## Results

In Target & Biomarker identification axis, from the 10 objectives outlined in SRA, those achieved were (1) identify and validate biological markers, tools and assays, (2) determinants of inter-individual variability, (3) understand the molecular mechanisms underlying the disease, (4) develop a platform of pre-clinical assays, and (5) develop systems models.

For the “innovative clinical trial paradigms” axis, the data applied two of the twelve objectives defined in SRA, especially (1) utilize innovative endpoints, trial designs, simulation and analytical approaches to devise new clinical trial paradigms and (2) develop innovative clinical endpoints.

In the innovative medicines axis, from the eleven objectives in SRA, those with results were: (1) identify new or alternative therapeutic concepts (targets) for treatment and prevention of disease, (2) develop novel therapeutic agents and disease prevention strategies, and (3) as implement new approaches for the development and production of biopharmaceuticals and tissue engineering.

Lastly, from the seven objectives in SRA, the data collected for maximizing patient-tailored adherence programmes address only one goal, namely develop patient-centered predictive models.

**Figure 2:**
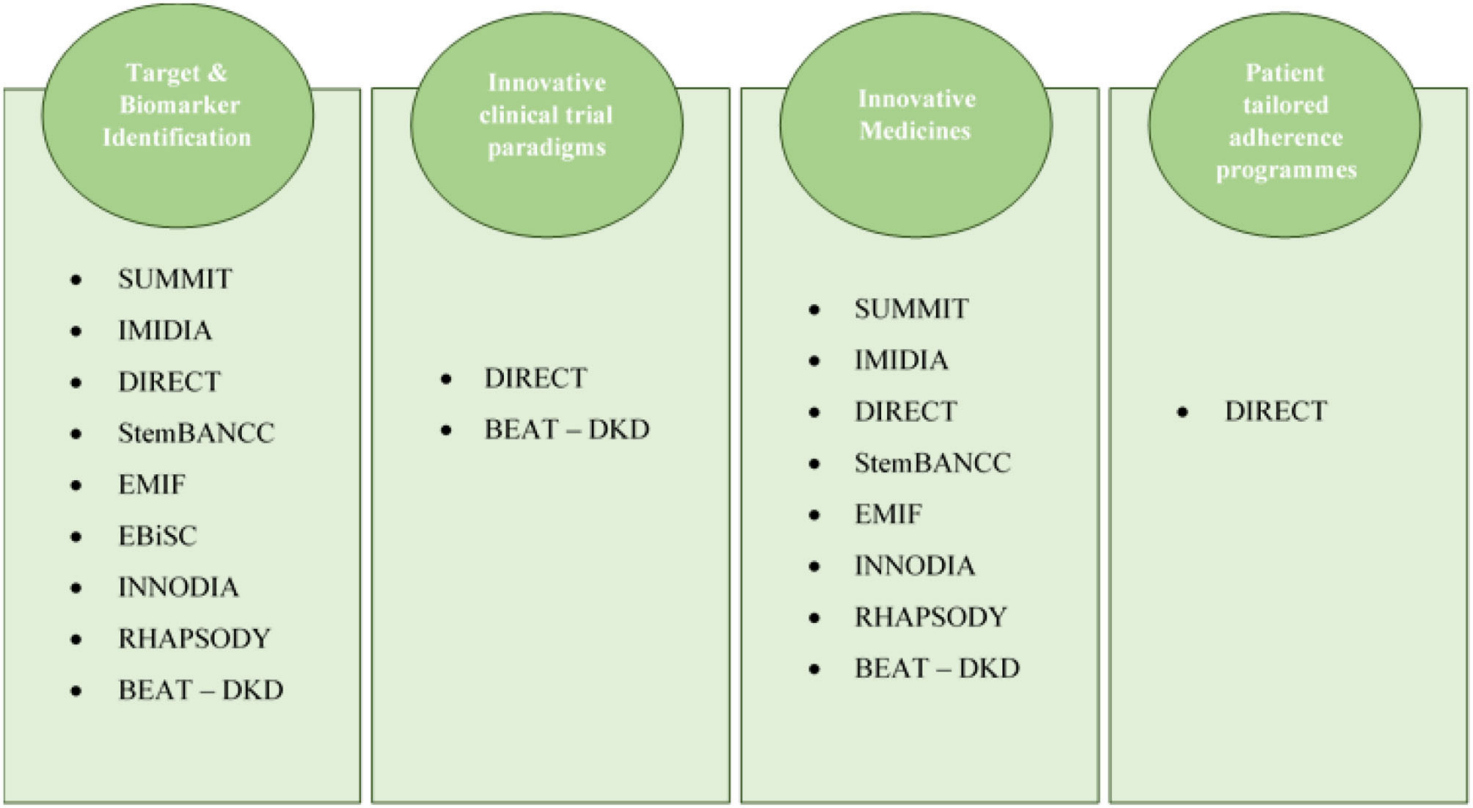
Projects' distribution according to their outcomes and the axes of the IMI2 programme.

In short, the outline of the projects with results in the research axes of the IMI2 programme, namely target & biomarker identification, innovative clinical trials paradigms, innovative medicines, and patient-tailored adherence programmes, is displayed in Figure 2.

No outputs were identified in LITMUS, Hypo-RESOLVE, IMI2PACT, and CARDIATEAM projects, as these were starting close to or during the literature search process.

Due to the quantity and diversity of data collected, we summarized the results obtained by each IMI funded-project in figures that are presented in Supplementary Material section.

A wide range of biomarkers have been identified for the onset of type 1 diabetes (14–24) by INNODIA; for risk prediction (14, 15, 17–21) and identification of patients at high-risk of type 2 diabetes (25) by SUMMIT, IMIDIA, DIRECT, and EMIF; for pancreatic β-cells function and protection by RHAPSODY (26, 27) and INNODIA (14, 16, 22–24); for hyperglycemia by RHAPSODY (28); for protection, prediction, initiation, progression, patient stratification, and medicine efficacy of diabetic kidney disease (DKD) (29, 30) by SUMMIT and BEAT-DKD (31–39); for development of cardiovascular disease (CVD) by SUMMIT (30, 40, 41); and for the development of diabetic retinopathy (DR) by SUMMIT (30, 42, 43).

Additionally, several novel tools were identified for diabetes, T1D, T2D, diabetic complications, and genetic research. The tools for diabetes intended to diagnose and monitoring disease progression [IMIDIA (44–49), DIRECT (50), and RHAPSODY (51)]. The tools designed for T1D were focused on monitor β-cell function, screen individuals at high risk, and select the more benefic intervention [INNODIA (15, 52, 53)]. For T2D, a new test was proposed to follow-up patients' insulin treatment need [EMIF (54, 55)]. Regarding diabetic complications, new tools were developed to enable the detection of patients at high risk of developing CVD and DR [SUMMIT (30, 56, 57)]. At last, in genetic research area, new tools were validated for the identification of single nucleotide polymorphism [SUMMIT (58–61) and StemBANCC (62, 63)].

Concerning the novel determinants of inter-individual variability, SUMMIT (43, 60, 64–72), IMIDIA (46, 49, 73–76), DIRECT (50, 77–82), EMIF (83–89), INNODIA (22, 23, 90–93), and RHAPSODY (27, 28, 94, 95) proposed a significant number of genetic markers for predisposition, initiation, identification, and progression of diabetes and its complications. Additionally, SUMMIT and DIRECT verified the influence of genetic factors in patients' medicine-response (96–101), and BEAT-DKD identified a non-genetic inter-individual therapeutic variability factor, i.e., NT-proBNP levels (37, 102, 103). DIRECT also confirmed the influence of gut composition (99, 100), age of diagnosis (50), year of diagnosis (104), and BMI factors (105) on the onset of diabetes, and EMIF showed the association with other factors such as ethnicity and metabolic health on T2D risk and development of complications (106). Moreover, two models of patient stratification were proposed, one by INNODIA for glycemic control in patients with T1D (107) and another by RHAPSODY and BEAT-DKD related with the identification of the patient's risk level for certain diabetic complications (108).

Novel relevant pathways were proposed to understand β-cell development and function [by IMIDIA (109–113), StemBANCC (114–119), and RHAPSODY (27)], type 1 diabetes (by INNODIA (16, 20, 23, 120–125), type 2 diabetes [by DIRECT (50), EMIF (85, 126–130), and Rhapsody (131)], CVD [SUMMIT (30, 132) and INNODIA (133)], DKD [BEAT-DKD (32, 36, 134–138)], DR [SUMMIT (30, 42, 43)], endometrial cancer risk [EMIF (139)], dementia [EMIF (140)], non-alcoholic fatty liver disease [EMIF (54, 55, 141–143)], anorexia or bulimia [INNODIA (125)], and attention-deficit hyperactivity disorder [INNODIA (125)].

In terms of pre-clinical studies, StemBANCC (114, 144, 145), EBiSC (146, 147), and IMIDIA (45, 49) proposed innovative iPSCs lines derived from diabetes and created their own databases; StemBANCC, INNODIA and RHAPSODY developed three catalogs, namely β-cell' Bi-DOCS (148), HLA-I peptidome of β-cells (21) and cis-eQTLs for T2D (149); IMIDIA (44–49), StemBANCC (144, 150), and RHAPSODY (151) established several protocols to improve the reliability of laboratory studies; and SUMMIT (30, 152, 153) and IMIDIA (46) developed new specific animal models.

Regarding systems models, two new *in silico* models were generated by SUMMIT (30), one for clinical complications in T1D and the other for aspirin action. In addition, BEAT-DKD proposed three models associated with DKD, namely the *Drosophila* nephrocyte to reveal mechanisms of podocyte function and glomerular diseases (154), the systems biology to better prediction of patient's medicine- response (155), and an *in-silico* analysis to identify compounds reversing a set of renal age-associated genes associated with the disease's progression (32).

For clinical trials, two novel design models were proposed, one by DIRECT and a second by BEAT—DKD: the Genotype-based Recall (GBR) (156) and “umbrella” or “platform” trials (157, 158), respectively. Regarding the innovative clinical endpoints, DIRECT validated a prediction model for T2D–DIRECT-DETECT, which may be used in the selection process in clinical trials (159, 160).

New potential therapeutic targets were suggested for the treatment of accelerated atherosclerosis in diabetic patients (161) (SUMMIT), for treating glomerular disease in T2D patients (36, 135, 162–164) (SUMMIT), for T2D patients with obesity (165) (EMIF), for counteracting hyperglycemia in individuals with T2D (166) (IMIDIA), for prevent or reverse β-cell loss [IMIDIA (167), StemBANCC (114, 116, 168, 169) and INNODIA (14, 90, 120, 121, 170–172)], and for diabetes with a focus on the use of new concepts such as epitranscriptome-based therapy by RHAPSODY (173), StemBANCC (114–116, 168, 169, 174), EMIF (86), and RHAPSODY (173). In terms of novel pharmacologic agents for T2D, SUMMIT developed the clinical trials of Aleglitazar (175), RAAS inhibitors (176), and supported the use of low-dose aspirin for the secondary prevention of cerebro-cardiovascular events (177).

Furthermore, DIRECT demonstrated the cardio-metabolic benefit of metformin (178), BEAT-DKD supported the clinical efficacy of SGLT2 inhibitors and GLP1R agonists in diabetic patients with DKD (179, 180), and StemBANCC established four different stem cell-based replacement treatments (181–183). For large-scale production, StemBANCC demonstrated that the continuous peristaltic pump-based circulation technology, in a hydraulically driven bioreactor, can be a potential 3D tool and a key in this process (184).

Lastly, it was established two patient-centered screening tools for T2D, more precisely the “palette” model, based on molecular taxonomy, and the DIRECT-DETECT prediction model, composed by glycaemic deterioration biomarkers (159, 160, 185).

## Discussion

**Figure 3:**
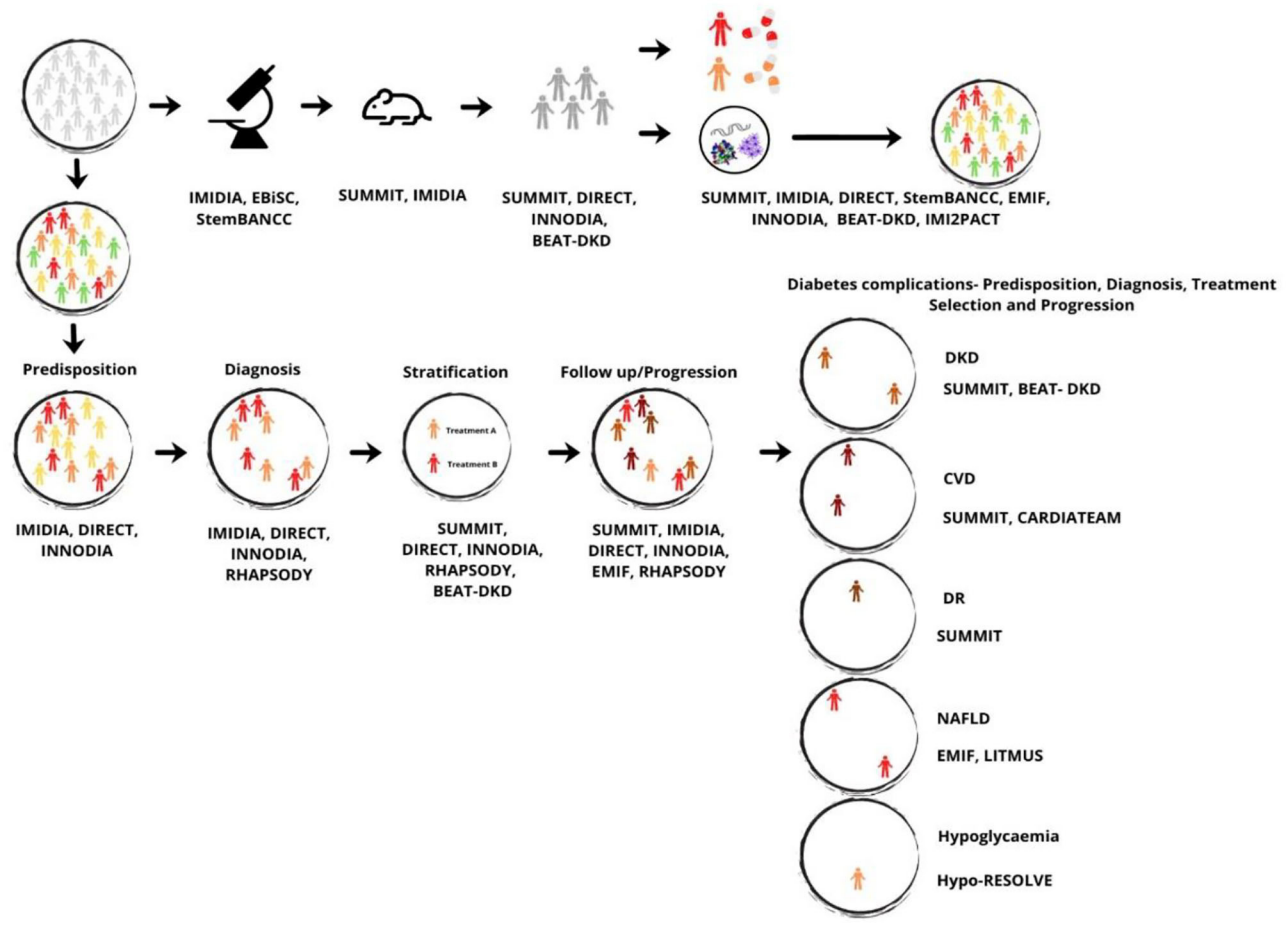
Integrated vision model for diabetes, based on the objectives and results of the IMI funded-projects. Color meaning: gray, general population; green, individuals at low risk of diabetes; yellow, individuals at medium risk of diabetes; orange, individuals at medium-high risk of diabetes; red, individuals at high risk of diabetes; dark orange, individuals at high risk of DKD; dark red, individuals at high risk of CVD; brown, individuals at high risk of DR; light red, individuals at high risk of NAFLD; and light orange, individuals at high risk of hypoglycaemia. BEAT-DKD, Biomarker Enterprise to Attack DKD Project; CARDIATEAM, Cardiomyopathy in Type 2 Diabetes Mellitus Project; CVD, Cardiovascular diseases; DIRECT, Diabetes Research on Patient Stratification Project; DKD, Diabetic Kidney Disease; DR, Diabetic Retinopathy; EBiSC, European Bank for induced pluripotent Stem Cells Project; EMIF, European Medical Information Framework Project; Hypo-RESOLVE, Hypoglycaemia, Redefining Solutions for Better Lives Project; IMIDIA, Improving Beta-cell Function and Identification of Diagnostic Biomarkers for Treatment Monitoring in Diabetes Project; INNODIA, Translational Approaches to Disease Modifying Therapy of Type 1 Diabetes: An Innovative Approach Toward Understanding and Arresting Type 1 Diabetes Project; LITMUS, Liver Investigation: Testing Marker Utility in Steatohepatitis Project; NAFLD, Non-Alcoholic Fatty Liver disease; RHAPSODY, Assessing Risk and Progression of Prediabetes and Type 2 Diabetes to Enable Disease Modification Project; StemBANCC, Stem Cells for Biological Assays of Novel Medicines and Predictive Toxicology Project; SUMMIT, Surrogate Markers for Micro- and Macro-Vascular Hard Endpoints for Innovative Diabetes Tools Project.

Based on the objectives of the IMI funded-projects and the results previously mentioned, we propose an integrated model addressing diabetes in its multiple dimensions, which includes two inter-dependent paths that should be executed in parallel, the first one being more scientific-oriented and the second one medical-oriented, as illustrated in Figure 3.

The scientific dimension would include the acquisition of more biological samples and genetic data and with the help of SUMMIT, IMIDIA, EBiSC, StemBANCC, EMIF, and IMI2PACT promote the research on β-cells as well as validate new biomarkers, genetic markers, patient stratification, discover more molecular mechanisms/pathways, and develop new treatments for T1D, T2D and diabetic complications. Besides, this approach aims at conducting clinical trials with more safety and efficacy endpoints through the application of those identified by SUMMIT, DIRECT, INNODIA, and BEAT-DKD to allow a marketing authorization of innovative medicines/therapeutics in a shorter time, less expensive and more focused on personalized medicine.

The medical dimension would include the use of predisposition markers developed by IMIDIA, DIRECT and INNODIA to identify people at higher risk of developing diabetes, with a particular interest in T2D, promoting the possibility of early intervention mainly in lifestyle habits, diet and physical exercise, and thus delaying the disease. Following the natural cycle of the disease, the objective would be to diagnose the recent-onset patients, through imaging technologies, tools and patient-centered models for clinicians developed by DIRECT, IMIDIA, INNODIA and RHAPSODY. Subsequently, the characterization of the subtype of patient and the treatment selection would be performed through the application of the DIRECT, INNODIA or RHAPSODY/BEAT-DKD stratification models and considering the inter-individual factors that impact the patient's response to the therapeutic agents identified by SUMMIT and DIRECT. The monitoring of disease progression would be possible in case of implementation of biomarkers, genetic markers and tools created by SUMMIT, IMIDIA, DIRECT, INNODIA, EMIF and RHAPSODY, with adaptations of pharmacological treatment dose or medicines changes in case of inadequate response. Additionally, with the use of biomarkers, genetic factors and tools developed by SUMMIT and BEAT-DKD, it would be possible to identify patients who during the progression of the disease, are more likely to develop diabetic complications, enabling to act in advance. With the application of imaging technologies developed by SUMMIT and EMIF and the information provided by BEAT-DKD, LITMUS, Hypo-RESOLVE, and CARDIATEAM, it would also be possible to predict the identification of patients with diabetic complications, especially diabetic nephropathy, diabetic retinopathy, cardiovascular disease, hypoglycemia, and non-Alcoholic Fatty Liver disease. Through the use of genetic factors and biomarkers developed by SUMMIT and BEAT-DKD, it would be desirable to select the best pharmacological treatment option according to the patient's characteristics and then monitor the follow-up to retard/stabilize its progression.

Summarizing, our integrated vision model supports a clinical model directed primarily and mainly at prevention, through the individual genetic and biological knowledge; as a first-line intervention, acting in the delay of diabetes onset; and in cases of diagnosis, to promote treatment according to the subtype of patients and monitor the progression of the disease. Only in this way, it will be possible to decrease the incidence and mortality rate of diabetes, provide an increase in the patient's quality of life, ensure sustainable people-centered health systems, and minimize direct and indirect diabetes-related costs in health systems.

Overall, it was found that the target & biomarker identification and innovative medicines axis have more published data. This was because these were major bottlenecks addressed by IMI 1 programme and included goals that corresponded to the key unmet needs in Diabetes during the programme's execution period 2008–2013.

Our literature review is subject to some limitations. When collecting the projects' data, we found a large gap in their publication's library, mainly SUMMIT, IMIDIA, and DIRECT projects. On SUMMIT website, there were only publications between 2010 and 2014, however, on the Cordis database, we found publications up to 2018. Similarly, although the IMIDIA website only included publications from 2011 and 2012, the Cordis database had articles until 2014. Regarding the DIRECT project, its website had only assembled the publications of the participating companies, all published before obtaining funding. Other limitations include the unsuccessful response from the project's coordinators and co-coordinators, and the CORDIS database was also not updated and complete.

## Conclusion

In order to reduce the incidence, the mortality rate, and the economic burden on healthcare systems, as well as to improve disease management, until October of 2019, IMI1 and IMI2 programmes funded 13 projects encompassing several bottlenecks identified for R&D and clinical practice in Diabetes area.

Taking into account the scientific production available by these projects, we prepared a joint vision model including two paths: one scientific-oriented and the other medical-oriented. The scientific dimension integrates the current knowledge regarding this disease, research tools, as well as clinical trial designs and endpoints to allow marketing authorization of new effective and safer medicines in a shorter time and less expensive. The medical dimension includes the application of predisposition markers (biological and genetic), diagnostic tools, stratification models, treatment selection, and monitoring the progression of the disease to prevent/delay the development of diabetic complications.

As IMI programmes fostered the enhancing of knowledge and the improvement of the medical practice (with better tools, medicines, and prediction models), being a big step for the implementation of personalized medicine, it is clear that this initiative has an important role in the scientific advances that have occurred in recent years.

In terms of future perspectives, the biggest bottleneck will be the implementation of the proposed joint vision model, or a similar one, into the clinical practice, although all IMI-funded projects highlight this trend as the only one able to provide an effective response in the treatment of chronic diseases, in particular diabetes, and there are already proves of shifts in the paradigm. Nevertheless, the involvement of key stakeholders, including patients, will always be essential to the success of this process.

## Author Contributions

MB was responsible for the conduct of the study, data extraction and analysis and drafted the manuscript. BS-L had the original idea for the manuscript and supervised study conduct. BS-L and CT participated in the collection of additional literature, contributed to the writing of the manuscript and revised it critically for important intellectual content. All authors read and approved the final manuscript.

## Conflict of Interest

The authors declare that the research was conducted in the absence of any commercial or financial relationships that could be construed as a potential conflict of interest.
