# Supplementary material for: Scientific Advances in Diabetes: The Impact of the Innovative Medicines Initiative
Source: Front Med (Lausanne). 2021 Jul 6;8:688438. doi: 10.3389/fmed.2021.688438 (PMC8290522; doi:10.3389/fmed.2021.688438)
Supplement: Supplementary file 1 [file Table_1.pdf]

frontiers

Detailed description of the projects and its objectives

Table 1 – Brief Description of IMI-funded projects in diabetes

Project	Call	Contribution	Duration	Grant number	Objectives
**SUMMIT:** Surrogate markers for micro- and macro-vascular hard endpoints for innovative diabetes tools (closed)	IMI 1 – Call 1	IMI Funding (14 654 559) EFPIA in kind (15 252 050) Other (4 905 472) Total Cost: 34 812 081€	01/11/2009 to 31/10/2015	115006	(1) Identify and characterize markers associated to diabetic nephropathy, diabetic retinopathy, and Lower Extremity Arterial Disease in both type 1 and type 2 diabetes as well as on cardiovascular disease in type 2 diabetes, to predict risks of developing the complications and monitor the effects of therapeutic interventions. (2) Develop knowledge, procedures, technologies and tools to make

					clinical trials testing of novel medications in diabetic complications shorter and more focused.
**IMIDIA:** Improving beta-cell function and identification of diagnostic biomarkers for treatment monitoring in diabetes<br><br>(closed)	IMI 1 – Call 1	IMI Funding<br><br>(8 060 760)<br><br>EFPIA in kind<br><br>(16 940 659)<br><br>Other<br><br>(2 445 590)<br><br>Total Cost:<br><br>27 447 009€	01/02/2010 to<br><br>30/09/2015	115005	(1) Deliver novel tools for the study of human beta-cell development, function and survival, their modulation by potential therapeutic compounds, and for *in vivo* beta-cell imaging.<br>(2) Deliver biomarkers for the diagnosis and prognosis of beta-cell failure and for monitoring diabetes progression and treatment.<br>(3) Deliver knowledge on novel molecular pathways and sites

					that control beta-cell life and death as well as mass and function.
**DIRECT:** Diabetes research on patient stratification<br><br>(closed)	IMI 1 – Call 3	IMI Funding<br><br>(21 388 643)<br><br>EFPIA in kind<br><br>(18 816 527)<br><br>Other<br><br>(6 278 957)<br><br>Total Cost:<br><br>46 484 127€	01/02/2012 to 31/07/2019	115317	(1) Identify the subtypes of type 2 diabetes patients.<br>(2) Identify and validate biomarkers associated with different subtypes of type 2 diabetes and different rates of disease progression.<br>(3) Determine the most appropriate treatments for each subtype of type 2 diabetes patients.
**StemBANCC:** Stem cells for biological assays of novel drugs and predictive toxicology<br><br>(closed)	IMI 1 – Call 4	IMI Funding<br><br>(26 000 000)<br><br>EFPIA in kind<br><br>(20 761 386)<br><br>Other<br><br>(8 249 094)<br><br>Total Cost:	01/10/2012 to 31/03/2018	115439	(1) Generate high-quality human induced pluripotent stem cell lines to study a range of chronic diseases (peripheral neuropathies, neurodegenerative disorders, neurodysfunctional disorders, diabetes)

		55 010 480€			and test for drug efficacy and safety. (2) Characterize iPS cell lines in terms of their genetic, protein, and metabolic profiles.
**EMIF:** European Medical Information Framework (closed)	IMI 1 – Call 4	IMI Funding (24 356 096) EFPIA in kind (24 354 503) Other (7 073 712) Total Cost: 55 784 311€	01/01/2013 to 30/06/2018	115372	(1) Develop a common information framework of patient-level data that will link up and facilitate access to diverse medical and research data source. (2) Identify predictors of metabolic complications in obesity. (3) Identify predictors of Alzheimer´s disease in the pre-clinical and prodromal phase.
**EBiSC:** European Bank for induced	IMI 1 – Call 8	IMI Funding (21 840 380)	01/01/2014 to	115582	(1) Establish a European iPS cell bank that will be

pluripotent Stem Cells<br>(closed)		EFPIA in kind<br>(7 167 072)<br>Other<br>(5 320 406)<br>Total Cost:<br>34 327 858€	31/12/2017		the 'go-to' resource for the characterisation, storage and distribution of high-quality iPS cells.
**INNODIA:** Translational approaches to disease modifying therapy of type 1 diabetes: an innovative approach towards understanding and arresting type 1 diabetes.<br>(ongoing)	IMI 2 – Call 1	IMI Funding<br>(17 630 000)<br>EFPIA in kind<br>(12 745 192)<br>Associated Partners<br>(9 164 968)<br>Other<br>(633 000)<br>Total Cost:<br>40 173 160€	01/11/2015 to 31/10/2022	115797	(1) Advance the understanding of type 1 diabetes and address the lack of tools and technologies that will allow clinicians to predict, evaluate and prevent the onset and progression of type 1 diabetes.<br>(2) Perform clinical intervention studies leading to novel therapies for preventing and curing type 1 diabetes.
**RHAPSODY:** Assessing risk	IMI 2 – Call 3	IMI Funding	01/04/2016 to	115881	(1) Understand the factors that drive the

and progression of prediabetes and type 2 diabetes to enable disease modification. (ongoing)		(8 130 000) EFPIA in kind (8 371 626) Other (2 189 500) Total Cost: 18 691 126€	31/03/2020		progression of pre-diabetes to diabetes and the deterioration of the condition of people with diabetes. (2) Develop novel biomarkers to refine diagnosis leading to better patient stratification, promote prevention, and support innovative drug discovery for personalized management of type 2 diabetes.
**BEAT-DKD:** Biomarker enterprise to attack DKD (ongoing)	IMI 2 – Call 5	IMI Funding (15 085 937) EFPIA in kind (13 360 968) Associated Partners (1 850 999) Total Cost: 30 297 904€	01/09/2016 to 31/08/2021	115974	(1) Provide a holistic systems medicine view of the pathogenesis DKD to identify targetable mechanisms and pathways underlying initiation and progression of DKD, applying a novel sub-

					classification of diabetes.<br>(2) Identify and validate biomarkers of disease progression and treatment responses representing the first steps towards precision medicine in the management of DKD.
**LITMUS:**<br><br>Liver Investigation: Testing Marker Utility in Steatohepatitis<br><br>(ongoing)	IMI 2 – Call 9	IMI Funding<br><br>(15 797 881)<br><br>EFPIA in kind<br><br>(24 180 663)<br><br>Other<br><br>(7 302 863)<br><br>Total Cost<br><br>47 281 407€	01/11/2017 to 31/10/2022	777377	(1) Develop, robustly validate and advance towards regulatory qualification biomarkers that diagnose, risk stratify and/or monitor NAFLD and NASH progression and fibrosis stage.<br>(2) Develop and validate imaging techniques that will allow doctors and researchers to rapidly and easily diagnose the severity of patients’

					disease and monitor changes in patients' livers.
**Hypo-RESOLVE:** Hypoglycaemia - REdefining SOLutions for better liVEs (ongoing)	IMI 2 – Call 10	IMI Funding (13 450 057) EFPIA in kind (10 316 000) Associated Partners (3 008 525) Total Cost: 26 774 583€	01/05/2018 to 30/04/2022	777460	(1) Provide researchers and clinicians with more validated data about the condition by creating a sustainable clinical database, by conducting studies to better understand the underlying mechanisms of hypoglycaemia, by conducting a series of statistical analyses to define predictors and consequences of hypoglycaemia, and by calculating the financial cost in European countries.
**IM2PACT:** Investigating mechanisms and	IMI 2 – Call 12	IMI Funding (9 000 000)	01/01/2019 to	807015	(1) Advance the understanding of the blood-brain

models predictive of accessibility of therapeutics into the brain		EFPIA in kind<br>(8 410 136)<br>Total Cost:<br>17 410 136€	31/12/2023		barrier to facilitate the development of more effective treatments for a range of neurological and metabolic disorders;<br>(2) Develop better models of the BBB so that researchers can study it more easily;<br>(3) Investigate the biology of the BBB in both health and disease, and the transport routes across it;<br>(4) Develops innovative systems capable of delivering medicines to the brain.
**CARDIATEAM:**<br>Cardiomyopathy in type 2 diabetes mellitus	IMI2 – Call 13	IMI Funding<br>(6 700 000)<br>EFPIA in kind<br>(6 000 000)	01/03/2019 to 29/02/2024	821508	(1) Determine how type 2 diabetes represents a central mechanism contributing to the pathogenesis and

(ongoing)		Other<br>(182 500)<br>Total Cost:<br>12 882 500€			progression of diabetic cardiomyopathy.<br>(2) Determine how distinct diabetic cardiomyopathy is from other forms of heart failure.
