# Supplementary material for: Scientific Advances in Diabetes: The Impact of the Innovative Medicines Initiative
Source: Front Med (Lausanne). 2021 Jul 6;8:688438. doi: 10.3389/fmed.2021.688438 (PMC8290522; doi:10.3389/fmed.2021.688438)
Supplement: Supplementary file 2 [file Table_2.pdf]

frontiers

List of DIRECT publications

Table 2 - List of DIRECT publications.

Nature of Communication	Title	Responsible Participant	Date	Target audience
**Publications**				
Peer Review Journal Article: Ahmad S et al. PLoS Genet. 2013;9(7):e1003607. doi: 10.1371/journal.pgen.1003607. Epub 2013 Jul 25. PubMed PMID: 23935507	Gene × physical activity interactions in obesity: combined analysis of 111,421 individuals of European ancestry	15/ULUND	25 Jul 2013	Diabetes researchers and practitioners
Peer Review Journal Article: Koivula et al. Diabetologia. *Provisionally accepted*	Discovery of biomarkers for glycaemic deterioration before and after the onset of type 2 diabetes: rationale and design of the epidemiological studies within the IMI DIRECT consortium	15/ULUND	Submitted Dec 2013	Diabetes researchers and practitioners
Peer Review Journal Article: Zhou et al.	Clinical and genetic determinants of progression of type 2 diabetes: A DIRECT Study	02/UNIVDUN	01 Nov 2013	Diabetes researchers and practitioners

Diabetes Care. 2013 Nov 1. [Epub ahead of print] PubMed PMID: 24186880				
Peer Review Journal Article: Franks et al. Diabetes Care. 2013 May;36(5):1413-21. doi: 10.2337/dc12-2211. Review. PubMed PMID: 23613601	Gene-environment and gene-treatment interactions in type 2 diabetes: progress, pitfalls, and prospects	15/ULUND, 02/UNIVDUN	May 2013	Diabetes researchers and practitioners
Rouskas et al. SOARD. Provisionally accepted	Weight loss independent association of TCF7L2 gene polymorphism with fasting blood glucose after Roux-en-Y Gastric Bypass in type 2 diabetes patients	26/CHRU LILLE	Submitted Dec 2013	Diabetes researchers and practitioners
Pasquali et al. Nature Genetics 2014 Jan 12 [Epub ahead of print]. PMID: 24413736.	Pancreatic islet enhancer clusters enriched in Type 2 diabetes risk-associated variants.	08/IDIBAPS, 19/UOXF	Jan 2014	Scientists
Peer Review Journal Article: Koivula et al. Diabetologia (PMID: 24695864)	Discovery of biomarkers for glycaemic deterioration before and after the onset of type 2 diabetes: rationale and design of the epidemiological studies within the IMI DIRECT consortium	ULUND	Published 2014 Apr	Diabetes researchers and practitioners
Manuscript: Journal for Diabetes Health Professionals in The Netherlands (in Dutch)	Will DNA be of help in treating type 2 diabetes patients?	LUMC	May	Health professionals

Journal article	Poveda A, Koivula RW, Ahmad S, Barroso I, Hallmans G, Johansson I, Renström F, Franks PW. Innate biology versus lifestyle behaviour in the aetiology of obesity and type 2 diabetes: the GLACIER Study. Diabetologia. 2015 Dec 1. [Epub ahead of print] PubMed PMID: 26625858.	ULUND	01 Dec 2015	Scientific / Clinical Community
GBR paper	A paper on the methodological features of genotype-based recall trial, which underpins potential clinical trial designs for Stage 2 of DIRECT (in review, Diabetes Care)	ULUND, UNIVDUN	10 Dec 2015	Scientific / Clinical Community
GBR website	A web tool to help design special randomized controlled trials for testing precision medicine hypotheses will be published in the above paper and will be of immediate value to DIRECT when designing the Stage 2 trials	ULUND, UNIVDUN	24 April 2015	Scientific / Clinical Community
Journal article	Statistical power considerations in genotype-based recall randomized controlled trials.	Atabaki-Pasdar N, Ohlsson M, Shungin D, Kurbasic A, Ingelsson E, Pearson ER, Ali A, Franks PW.	Nature Sci Rep. 2016 Nov 25;6:37307. doi: 10.1038/srep37307. PMID: 27886175	Scientific community
Journal article	Causal inference in obesity research.	Franks PW, Atabaki-Pasdar N.	J Intern Med. 2016 Dec 8. doi: 10.1111/joim.12577. [Epub ahead of print] Review. PMID: 27933671	Scientific community
Journal article	Lifestyle and precision diabetes medicine: will genomics help optimise the prediction, prevention and treatment of type 2 diabetes through lifestyle therapy?	Franks PW & Poveda A.	Diabetologia Epub Jan 2017	Scientific community

Journal article	Exposing the exposures in type 2 diabetes.	Franks PW & McCarthy MI	Science. 354(6308):69-73. 2016	Scientific community
Journal article	Putting the genome in context: gene-lifestyle interactions in type 2 diabetes.	Franks PW & Pare G.	Curr Diab Rep. 6(7):57. 2016	Scientific community
Journal article	Sustained influence of metformin therapy on circulating glucagon-like peptide-1 levels in individuals with and without type 2 diabetes.	Preiss D, Dawed A, Welsh P, Heggie A, Jones AG, Dekker J, Koivula R, Hansen TH, Stewart C, Holman RR, Franks PW, Walker M, Pearson ER, Sattar N DIRECT consortium group	Diabetes Obes Metab. 2017 Mar;19(3):356-363. doi: 10.1111/dom.12826. PMID: 27862873.	Scientific community
Journal article	Predicting glycated hemoglobin levels in the non-diabetic general population: Development and validation of the DIRECT-DETECT prediction model - a DIRECT study.	Rauh SP, Heymans MW, Koopman AD, Nijpels G, Stehouwer CD, Thorand B, Rathmann W, Meisinger C, Peters A, de Las Heras Gala T, Glümer C,	PLoS One. 2017 Feb 10;12(2):e0171816. doi: 10.1371/journal.pone.0171816. PMID: 28187151	Scientific community

		Pedersen O, Cederberg H, Kuusisto J, Laakso M, Pearson ER, Franks PW, Rutters F, Dekker JM.		
Journal article	Personalized medicine in diabetes: the role of 'omics' and biomarkers.	Pearson ER. /UNIVDUN	Diabet Med. 2016 Jun;33(6):712-7. doi: 10.1111/dme.13075. Review.PMID: 26802434	Scientific community
Journal article	Painting a new picture of personalised medicine for diabetes	Mark McCarthy / UOXF	Diabetologia 2017 Feb 7 doi: 10.1007/s00125-017-4210-x. [Epub ahead of print]	Scientific community
Journal article	A Genome-Wide Association Study of IVGTT-Based Measures of First-Phase Insulin Secretion Refines the Underlying Physiology of Type 2 Diabetes Variants	Wood AR, Diabetes Research on Patient Stratification (DIRECT), et al	Diabetes. 2017 Aug;66(8):2296-2309. doi: 10.2337/db16-1452.	Scientific community
Journal article	Integrative network analysis highlights biological processes underlying GLP-1 stimulated insulin secretion: A DIRECT study	Gudmundsdottir V, Pedersen HK, Allebrandt KV, Brorsson C, van	PLoS One. 2018 Jan 2;13(1):e0189886. doi: 10.1371/journal.	Scientific community

		Leeuwen N, Banasik K, Mahajan A, Groves CJ, van de Bunt M, Dawed AY, Fritsche A, Staiger H, Simonis-Bik AMC, Deelen J, Kramer MHH, Dietrich A, Hübschle T, Willemsen G, Häring HU, de Geus EJC, Boomsma DI, Eekhoff EMW, Ferrer J, McCarthy MI, Pearson ER, Gupta R, Brunak, 't Hart LM.	pone.0189886. PubMed PMID: 29293525.	
Journal article	Metabolite ratios as potential biomarkers for type 2 diabetes: a DIRECT study	Molnos S, Wahl S, Haid M et al.	Diabetologia. 2018 Jan;61(1):117-129. doi: 10.1007/s00125-017-4436-7	Scientific community
Journal article	Long-Term Stability of Human Plasma Metabolites during Storage at −80 °C	Haid M et al.	J. Proteome Res., 2018, 17 (1), pp 203–211	Scientific community

Journal article	Mass spectrometry based qualification of antibodies for plasma proteomics	Claudia Fredolini, Sanna Bystrom et al.	https://www.biorxiv.org https://doi.org/10.1101/158022	Scientific community
