# Supplementary material for: Scientific Advances in Diabetes: The Impact of the Innovative Medicines Initiative
Source: Front Med (Lausanne). 2021 Jul 6;8:688438. doi: 10.3389/fmed.2021.688438 (PMC8290522; doi:10.3389/fmed.2021.688438)
Supplement: Supplementary file 3 [file Data_Sheet_1.pdf]

List of Abbreviations

List of Abbreviations

ABACUS – Algorithm based on a Bivariate Cumulative Statistic

ADHD – Attention-Deficit Hyperactivity Disorder

BCAA – Branched-Chain Amino Acids

BEAT-DKD – Biomarker Enterprise to Attack DKD Project

BiPSC – β-cell derived iPSCs

Bi-DOCS – BiPSC Specific Differential Open Chromatin Sites

BMI – Body Mass Index

BoNB – Bag of Naïve Bays

BOSS – Binary Outcome Stochastic Search

CARDIATEAM – Cardiomyopathy in Type 2 Diabetes Mellitus Project

CORDIS – Community Research and Development Information Service

CVD – Cardiovascular diseases

CYP2C9 – Cytochrome P450 2C9

DIRECT – Diabetes Research on Patient Stratification Project

DKD – Diabetic Kidney Disease

DM – Diabetes Mellitus

DR – Diabetic Retinopathy

EBiSC – European Bank for induced pluripotent Stem Cells Project

EC – European Community

EFPIA – European Federation of Pharmaceutical Industries and Associations

eGFR – Estimated Glomerular Filtration Rate

EMIF – European Medical Information Framework Project

EU – European Union

GADA – Glutamic Acid Decarboxylase Autoantibodies

GBR – Genotype-based Recall

GLP1R – GLP1 Receptor

HbA_{1c} – Glycated Haemoglobin

HNB – Hierarchical Naïve Bayes

HPSCs – Human Pluripotent Stem Cells

Hypo-RESOLVE – Hypoglycaemia - Redefining Solutions for Better Lives Project

IMI – Innovative Medicines Initiative

IMI1 – First Innovative Medicines Initiative programme

IMI2 – Second Innovative Medicines Initiative programme

IM2PACT – Investigating Mechanisms and Models Predictive of Accessibility of Therapeutics into the Brain Project

IMIDIA – Improving Beta-cell Function and Identification of Diagnostic Biomarkers for Treatment Monitoring in Diabetes Project

INNODIA – Translational Approaches to Disease Modifying Therapy of Type 1 Diabetes: An Innovative Approach Towards Understanding and Arresting Type 1 Diabetes Project

iPS – Induced Pluripotent Stem

iPSCs – Induced Pluripotent Stem Cells

LITMUS – Liver Investigation: Testing Marker Utility in Steatohepatitis Project

MODY – Maturity-Onset Diabetes of the Young

NAFLD – Non-Alcoholic Fatty Liver disease

NASH – Non-alcoholic Steatohepatitis

NFAT – Nuclear Factor of Activated T-cells

OCT – Optical Coherence Tomography

PPP – Public and Private Partnership

RAAS – Renin-Angiotensin-Aldosterone System

RC – Redifferentiation Cocktail

RHAPSODY – Assessing Risk and Progression of Prediabetes and Type 2 Diabetes to Enable Disease Modification Project

R&D – Research and Development

SNP – Single nucleotide polymorphism

SRA – Strategic Research Agenda

StemBANCC – Stem Cells for Biological Assays of Novel Medicines and Predictive Toxicology Project

SUMMIT – Surrogate Markers for Micro- and Macro-Vascular Hard Endpoints for Innovative Diabetes Tools Project

T1D – Type 1 diabetes

T2D – Type 2 diabetes

UPSA – Ultrasound Plaque Structure Analysis

VEGFA – Vascular Endothelial Growth Factor

WHO – World Health Organization
