# Supplementary material for: Scientific Advances in Diabetes: The Impact of the Innovative Medicines Initiative
Source: Front Med (Lausanne). 2021 Jul 6;8:688438. doi: 10.3389/fmed.2021.688438 (PMC8290522; doi:10.3389/fmed.2021.688438)
Supplement: Supplementary file 4 [file Data_Sheet_2.pdf]

The results obtained by each IMI funded-project

Target & Biomarker Identification

Novel Biomarkers

- T2D (e.g. no evidence for the association of lower adiponectin levels with T2D)
- DKD (e.g. inverse relationship between serum UMOD and disease's risk)
- CVD (e.g N-terminal prohormone B-type natriuretic peptide and Apolipoprotein CIII improved prediction of CVD)
- DR (e.g. VEGFA, NFAT, caspase-3 activation, and advanced glycation end products were associated with development of DR)

Platform of pre-clinical assays

- DKD (e.g. glomerular expression profile database for T1D and T2D mouse models)
- CVD (e.g. new animal models such as SUR1xIGFII)
- DR (e.g. development of Akita mouse model)

Target & Biomarker Identification

Novel Tools

- CVD (e.g. two diagnostic tools were developed, more precisely the Ultrasound Plaque Structure Analysis (UPSA)-system that enables the assessment of atherosclerosis plaque structure, and the tracer 18F-fluoromethylcholine (18F-FMCH) for the evaluation of atherosclerotic vascular inflammation in diabetes)
- DR (e.g it was developed the Optical Coherence Tomography (OCT) that improve prediction of diabetic retinopathy)
- Genetic research (e.g four new tools were validated, specifically Hierarchical Naïve Bayes (HNB), Binary Outcome Stochastic Search (BOSS), 'Bag of Naïve Bays' (BoNB) and Algorithm based on a Bivariate Cumulative Statistic (ABACUS))

Develop systems models

Two *in silico* models were generated, more precisely (i) Dynamic Bayesian Network model for long-term simulation of clinical complications in T1D, and (ii) *In silico* model of aspirin action of platelet and megakaryocyte cyclooxygenase-1.

SUMMIT

Target & Biomarker Identification

Genetic markers/factors

- DM (e.g. glycaemic response to metformin varie in 34% according to the hereditable phenotype for the absolute reduction in HbA1c)
- DKD (e.g. twenty-one SNPs were associated with risk of this disease, through mechanisms including microalbuminuria and impairment/decrease of estimated glomerular filtration rate)
- CVD (e.g. three known coronary artery calcification were associated with its development in patients with T2D)

Molecular mechanisms underlying disease

- CVD (e.g. it was suggested that cardiovascular events might occur at a lower atheroma burden in diabetes)
- DR (e.g. a novel phenotype form of retinopathy was identified which presented alterations in retinal morphology)

Innovative Medicines

Identify new or alternative therapeutic concepts (targets) for treatment and prevention of disease

- T2D and CVD (e.g. for the treatment of accelerated atherosclerosis in diabetes, SUMMIT proposed as a potential target the inhibition of the Nuclear Factor of Activated T-cells signalling)
- T2D and DKD (e.g for treating of glomerular disease in T2D patients the pathways proposed were reducing YAP activity, anti-miR-92a options, such as anti-STAT3, partial mTOR inhibition, PI3K-Akt, and Raf-MEK-ERK signalling)

Develop novel therapeutic agents and disease prevention strategies

- T2D (e.g. aleglitazar was under development by Hoffmann–La Roche for the treatment of patients with T2D)
- T2D and DKD (e.g renin-angiotensin-aldosterone-system inhibitors evidenced in clinical trials that slowed the onset of T2D and reduced the risk of renal complications)

Figure 1 - Summary of SUMMIT's project innovation.

Target & Biomarker Identification

Novel Biomarkers

- T2D (e.g. high free fatty acids levels were associated with the risk of T2D)

Genetic markers

- T2D (e.g. genetic markers were associated with T2D predisposition, development and progression, such as the rare mutation p.G1117E in the PASK gene in a single MODY family)

Target & Biomarker Identification

Novel tools

- Diabetes (e.g. it was developed a manganese-enhanced high field magnetic resonance imaging tool suitable for diagnostic and monitoring diabetes progression)

Molecular mechanisms underlying disease

- β-cell protection (e.g. monounsaturated fatty acids exhibited opposite effects and counteracted the toxic effects of glucolipotoxicity)
- β-cell apoptosis (e.g. ELOVL2 downregulation significantly increased β-cell apoptosis induced by glucolipotoxicity)

IMIDIA

Target & Biomarker Identification

Platform of pre-clinical assays

- Biobank (e.g. it was produced an EndoC-BH1 cell line that expressed many specific β-cell markers)
- Animal models (e.g. It was created a model with pancreatic alpha cell-specific deletion of Tcf7l2, i.e. Tcf7l2AKO mice)
- Laboratory Protocol (e.g. it was published a protocol for islets isolation from human pancreatic tissue obtained from type 2 diabetes and non-diabetic patients)

Innovative Medicines

Identify new or alternative therapeutic concepts (targets) for treatment and prevention of disease

- New targets for diabetes (e.g activation of glucokinase, a glucose sensor in β-cells, for counteracting hyperglycaemia in individuals with type 2 diabetes)
- New concepts for diabetes (e.g. reprogram β-cells to insulin production after extreme β-cell loss)

Figure 2 - Summary of IMIDIA's project innovation.

Target & Biomarker Identification

Novel Biomarkers

- T2D (e.g. valine to phosphatidylcholine acyl-alkyl C32:2 metabolite was proposed for identification of individuals at high-risk)

Novel tools

- Diabtes (e.g. GADA test was proposed for prediction of diabetes progression since individuals with GADA-positive progress approximately two times faster)

Molecular mechanisms underlying disease

- T2D (e.g. the progression of T2D is mediated by genetic factors, glucolipotoxicity, endoplasmic reticulum, and oxidative stress)

Target & Biomarker Identification

Genetic markers

- T2D (e.g. it was evidenced that genetic factors associated with T2D predisposition are different from those that cause their progression)
- Treatment (e.g. patients with HNF1A mutations respond better to sulfonylureas than metformin, and cytochrome P450 2C9 (CYP2C9) *2 and *3 variation change sulphonylurea efficacy)

Other individual factors

- Gut composition (e.g. microbiome alterations induced by metformin may promote intolerance and affect efficacy)
- Age (e.g. the individuals diagnosed younger than 50 years of age progress rapidly compared with individuals diagnosed over the age of 70).

Sex (e.g. women were independently associated with rapid progression to the requirement of insulin treatment).

- Weight (e.g. individuals with normal weight at diagnosis are likely to be βcell-deficient and progress rapidly to insulin requirement, and those who are particularly adipose are likely to be markedly insulin resistant)
- Year (e.g. maternal obesity during pregnancy raises the offspring's risk of type 2 diabetes)

DIRECT

Innovative clinical trial paradigms

Utilise innovative endpoints, trial designs, simulation and analytical approaches

- A novel design model for clinical trials, genotype-based recall (GBR), exhibited to have a higher power to observe gene-drug and gene-lifestyle interactions than same-sized conventional randomized clinical trials, exceptionally when the sampling frame is too small to identify minor alleles and the recalled groups are not sufficiently distinc.

Develop innovative clinical endpoints

- DIRECT-DETECT was proposed to be used in the selection process in clinical trials. This is a prediction model based on biomarkers for glycaemic deterioration before and after the onset of type 2 diabetes.

Innovative Medicines

Develop novel therapeutic agents and disease prevention strategies

- T2D (e.g. Data supported that metformin may provide cardio-metabolic benefit, even in a non-diabetic population and beyond reducing the risk of developing T2D)

Patient tailored adherence programmes

Develop and apply patient-centred predictive models

It was established two screening tools for T2D, namely (i) the 'palette' model that is centred on a molecular taxonomy, which contributes to diabetes risk and progression, and (ii) the DIRECT-DETECT prediction model that is based on glycaemic deterioration biomarkers that allows the identification of non-diabetic individuals who are at high risk and prediction of disease progression.

Figure 3 - Summary of DIRECT's project innovation.

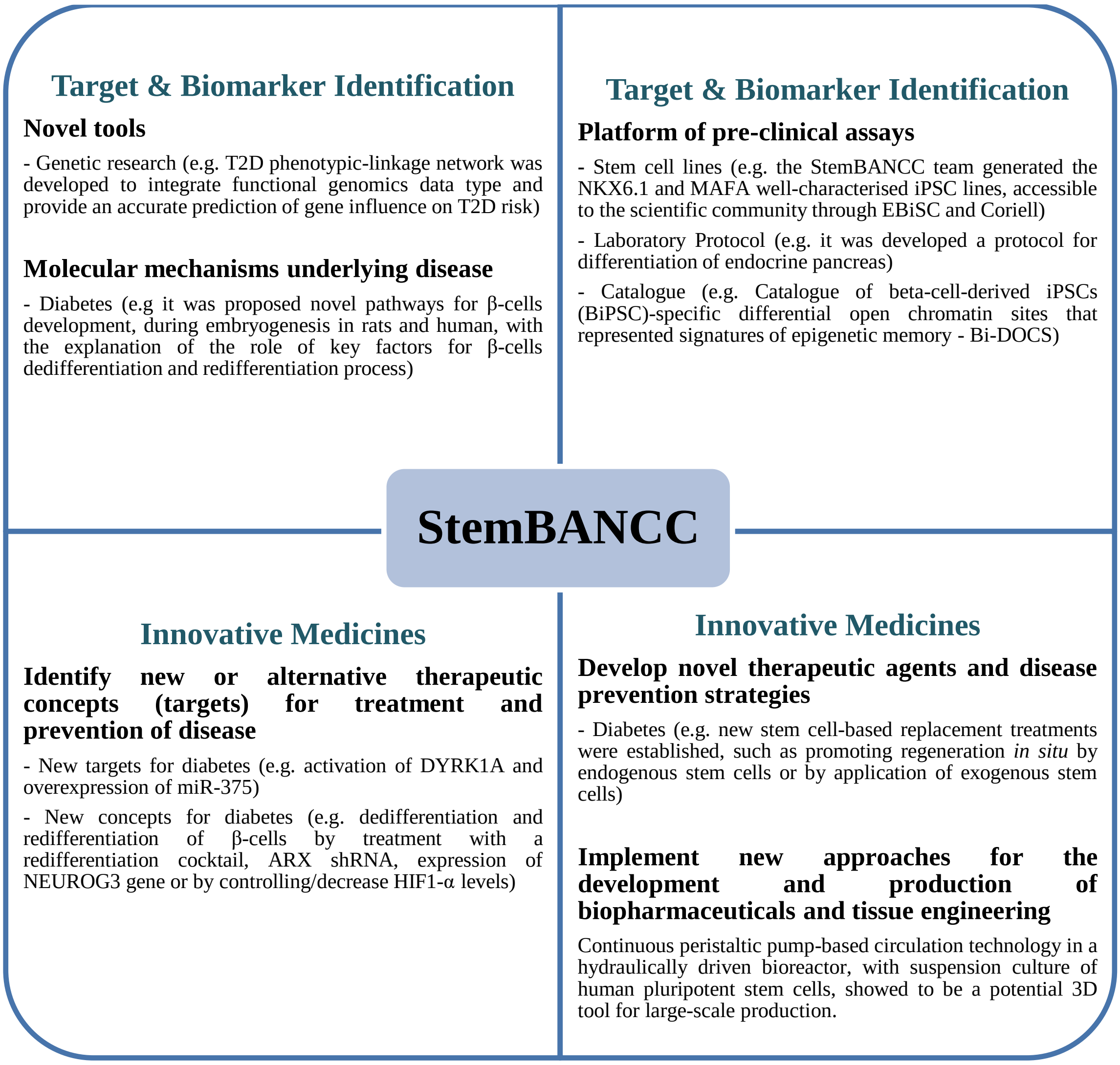

Figure 4 - Summary of StemBANCC's project innovation.

Target & Biomarker Identification

Novel biomarkers

- T2D (e.g. several biological markers were suggested to predict the risk of type 2 diabetes, more precisely gamma-glutamyl transpeptidase, plasma mannose, plasma metabolites, and adiponectin)

Novel tools

- T2D (e.g. the “Lipid triplet test” was proposed to follow-up T2D patients with insulin treatment. This tool predicts the amount of liver fat and intendeds to control hepatic glucose production)

Target & Biomarker Identification

Genetic markers

- Diabetes (e.g. some genetic mutations were associated with the increase of diabetes risk, namely PCYT1A mutations, NSMCE2 mutations, DNA CpG methylation in SAT and VAT, loss-of-function mutations of PPM1K, and PIK3R1 mutations)

Other inter-individual variability factors

- Ethnic (e.g. south asians have a greater prevalence of type 2 diabetes than do the background populations of the countries to which they moved, and the neonates seem to be more insulin resistant than white Europeans throughout childhood)

EMIF

Target & Biomarker Identification

Molecular mechanisms underlying disease

- T2D (e.g. patients with NAFLD associated with TM6SF2 variant which encodes Glu167Lys (E167K) metabolically silent were suggested to had a decrease risk to develop T2D)
- Dementia (data supported that diabetes was a risk factor of dementia by two potential pathways: dietary methyl-glyoxyl levels and downstream of SIRT1)
- Endometrial cancer (T2D was associated with endometrial cancer risk because insulin decreases the levels of sex hormone-binding globulin by inhibiting its production in the liver, which consequently, induces endometrial cell proliferation and decrease apoptosis, resulting in increased endometrial cancer risk)
- NAFLD/NASH (e.g. Patients with T2D have a 2-fold higher risk of NAFLD/NASH.)

Innovative Medicines

Identify new or alternative therapeutic concepts (targets) for treatment and prevention of disease

- New target for diabetes (e.g. modulation of BCAA metabolism)
- New target for T2D patients with obesity (e.g. upregulation of adipose tissue CIDEA mRNA levels)

Figure 5 - Summary of EMIF's project innovation.

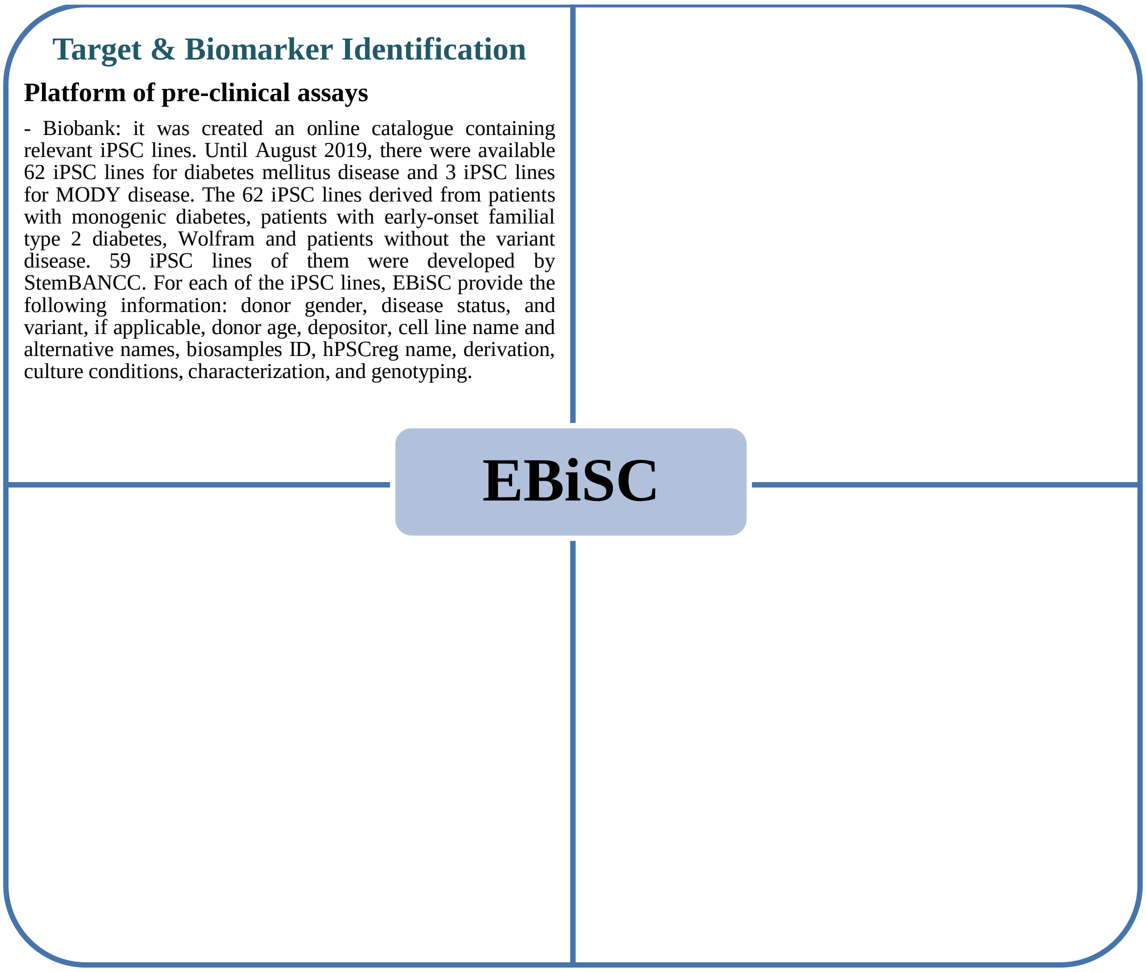

Figure 6 - Summary of EBiSC's project innovation.

Target & Biomarker Identification

Novel Biomarkers

- T1D (e.g. new β-cell biomarkers were identified, such as DPP6, FXYD2γa, PDL1, GAD, IA-2, and ZnT8 antibodies)

Novel tools

- T1D (e.g. the Dried Blood Spots method was developed with the purpose of measure C-peptide in children and adolescents recently diagnosed and to monitor β-cell function over the time at T1D risk individuals)

Platform of pre-clinical assays

- Catalogue (e.g. a first catalogue of the HLA-I peptidome of human β-cells, using an immortalized β-cell line naturally expressing the most prevalent HLA-A2 variant)

Target & Biomarker Identification

Genetic markers

- T1D (e.g. some genes were with the risk/development of T1D, such as HLA-DR3-DQ2 haplotype, dysregulation of miR-25-3p, and DEXI)

Other inter-individual variability factors

- Age (e.g. age was shown to have a profound impact on T1D since immune interventions exhibited greater effects in children compared to adults)

Models of patient stratification

- It was established five groups with distinct trajectories of glycaemic control (HbA1c) in children from age 8 and young adults with T1D. Groups differed by self-care with a lower frequency of self-monitoring of blood glucose in subjects with HbA1c deterioration and higher insulin dose among patients with HbA1c increase.

INNODIA

Target & Biomarker Identification

Molecular mechanisms underlying disease

- T1D (e.g. lifestyle interventions, especially exercise training programs, prevented, delayed or attenuated loss of functional β-cell mass in T1D, in part via activation of the transcription factor STAT3 (partially via IL-6) in β-cells and consequently ERK1/2 activation)
- T1D and CVD (e.g. in type 1 diabetes girls, oral contraceptives were associated with higher cardiovascular risk and worse metabolic control)
- T1D and aorexia/bulimia (e.g. it was observed that anorexia and bulimia were more prevalent in girls with type 1 diabetes, although clinically less serious eating disorders were observed in boys)
- T1D and Attention-deficit hyperactivity disorder (ADHD) (e.g. it was observed that ADHA was more common in boys with T1D)

Innovative Medicines

Develop novel therapeutic agents and disease prevention strategies

- New concept for diabetes (e.g. dedifferentiation and redifferentiation of β-cells through PolyI:C)
- New targets to prevent or reverse β-cell loss (e.g. prevent GRP78 surface translocation or suppress the GRP78 receptor signalling)

Figure 7 - Summary of INNODIA's project innovation.

Target & Biomarker Identification

Novel Biomarkers

- β-cells (e.g. some biomarkers were proposed for β-cells, namely transcription factor MondoA, MAF, and PAX6)

- Hyperglycaemia (e.g. the hyperglycaemia biomarkers suggested were SLC2A2, FFAR4, TMEM37, TMEM27, and ALDOB)

Genetic markers

- Diabetes (e.g. it was suggested that loss-of-function mutations in TRMT10A were a monogenic cause of early-onset diabetes during adolescence or young adulthood)

- T2D (e.g. some genetic markers were associated with increased susceptibility to type 2 diabetes, such as SNPs near CDC123 and CDKN2Agenes, SNPs in CDKAL1, SNPs in TRIT1, and SNPs in mt-tRNA)

- β-cells (e.g. the discovered genetic markers associated with β-cell dedifferentiation were TNFRSF11B gene, SOX9 gene, and NEUROG3 gene)

Target & Biomarker Identification

Models of patient stratification (RHAPSODY / BEAT-DKD)

Identification of five subtypes of patients with different risk levels for certain complications associated with diabetes. Cluster 1 was characterised by early-onset disease, relatively low BMI, poor metabolic control, insulin deficiency, presence of GADA and was labelled as severe autoimmune diabetes; Cluster 2 was categorised as severe insulin-deficient diabetes, with GADA negative, low age at onset, relatively low BMI, low insulin secretion and poor metabolic control; Cluster 3 was characterised as severe insulin-resistant diabetes, with insulin resistance and high BMI; Cluster 4 was labelled by obesity but not by insulin resistance and as mild obesity-related diabetes; and Cluster 5 was categorised as mild age-related diabetes, with older patients and similar to cluster 4, only with modest metabolic derangements. Patients in group 2 ('severe insulin-deficient diabetes') are at the greatest risk of eye disease, while patients in group 3 ('severe insulin-resistant diabetes') have the highest incidence of kidney damage.

RHAPSODY

Target & Biomarker Identification

Novel tools

- Diabetes (e.g. it was developed a machine learning developed by RHAPSODY to predict the glucose tolerance status by 22 baseline variables obtained at fasting blood acquisition)

Molecular mechanisms underlying disease

- β-cells (e.g. a novel pathway to induce β-cell dedifferentiation was suggested through the binding of FGF2 to FGF receptors 1c (FGFR1c), which induced the expression of SOX9, NEUROG3, and gastrin).

- T2D (e.g. amylin and nucleobindin–1 were found to inhibit insulin fibrillation, attenuated fibril-induced cell toxicity, and proposed to act in the control of amyloidogenesis in type 2 diabetes)

Target & Biomarker Identification

Platform of pre-clinical assays

- Laboratory Protocol (e.g. it was developed a protocol for AMPK function study in mouse and human pancreatic islets)

- Catalogue (e.g. it was created a catalogue of cis-eQTLs with relevance to T2D and HbA_{1c} levels)

Innovative Medicines

Identify new or alternative therapeutic concepts (targets) for treatment and prevention of disease

- New concept for diabetes (e.g. the use of tRNA fragments through epitranscriptome-based therapy)

Figure 8 - Summary of RHAPSODY 's project innovation.

Target & Biomarker Identification

Novel Biomarkers

- DKD prognosis (e.g. it was identified a set of 33 compounds with the potential of reversing the expression signature of renal age-associated DKD prognosis genes)

- DKD initiation (e.g. extracellular vesicles, i.e. urinary extracellular vesicles, was validated as a predictor of eGFR decline levels in patients with type 2 diabetes)

- Treatment selection (e.g. NT-proBNP could help to identify individuals who may not respond to dual RAAS inhibition with aliskiren and who may benefit from diuretic treatment or dietary sodium-lowering)

- Neuroprotective markers (e.g. a set of neuroprotective markers for DKD was established)

Develop system models

- DKD (e.g. it was settled an *in-silico* analysis approach to identify compounds reversing a set of renal age-associated genes significantly associated with DKD progression)

Target & Biomarker Identification

Inter-individual variability factors

- T2D (e.g. poor responders to renin angiotensin aldosterone system inhibition initiation in patients with type 2 diabetes, treated in primary care, had the highest risk of cardiovascular events and showed large variability in urinary albumin creatinine ratio)

Models of patient stratification (RHAPSODY / BEAT-DKD)

Identification of five subtypes of patients with different risk levels for certain complications associated with diabetes. See the figure related to RHAPSODY project.

BEAT-DKD

Innovative clinical trial paradigms

Utilise innovative endpoints, trial designs, simulation and analytical approaches

It was proposed a change in the trial design with medicines to DKD in T2D patients, selecting patients with a positive surrogate marker response to the medicine into trials and deselecting patients with a negative surrogate marker response. This claim was supported by two main reasons, firstly the possibility of trials be smaller, shorter, or both, and thus potentially less expensive, and secondly the non-responders patients will not be exposed to a medicine that is unlikely to benefit them. To help with this issue, it was proposed the concept of "basket" or "umbrella" trials, or more commonly called "platform" trials, that involves assessing the effects of multiple interventions on one or more conditions, using modern adaptive designs and statistical approaches, such as master trial protocol.

Innovative Medicines

Develop novel therapeutic agents and disease prevention strategies

- DKD (e.g. SGLT2 inhibitors and GLP1R agonists showed clinical efficacy in the treatment of DKD. SGLT2 inhibitors (such as canagliflozin) slowed the progression of DKD and GLP1R agonists prevented worsening of albuminuria)

Figure 9 - Summary of BEAT-DKD's project innovation.
